# Path integration and optic flow in flying insects: a review of current evidence

**DOI:** 10.1007/s00359-025-01734-9

**Published:** 2025-03-07

**Authors:** Martin Egelhaaf, Jens P. Lindemann

**Affiliations:** Neurobiology, Bielefeld University, Universitätsstraße 25, 33615 Bielefeld, Germany

**Keywords:** Navigation, Goal finding, Path integration, Optic flow, Distance estimation

## Abstract

Path integration is a key navigation mechanism used by many animals, involving the integration of direction and distance of path segments to form a goal vector that allows an animal to return directly to its starting point. While well established for animals walking on solid ground, evidence for path integration in animals moving without ground contact, such as flying insects, is less clear. The review focuses on flying Hymenoptera, particularly bees, which are extensively studied. Although bees can use flight distance and direction information, evidence for genuine path integration is limited. Accurately assessing distance travelled is a major challenge for flying animals, because it relies on optic flow—the movement of visual patterns across the eye caused by locomotion. Optic flow depends on both the animal’s speed and the spatial layout of the environment, making it ambiguous for precise distance measurement. While path integration is crucial for animals like desert ants navigating sparse environments with few navigational cues, we argue that flying Hymenopterans in visually complex environments, rich in objects and textures, rely on additional navigational cues rather than precise path integration. As they become more familiar with an environment, they may iteratively refine unreliable distance estimates derived from optic flow. By combining this refined information with directional cues, they could determine a goal vector and improve their ability to navigate efficiently between key locations. In the case of honeybees, this ability also enables them to communicate these refined goal vectors to other bees through the waggle dance.

## Introduction

The ability to navigate back to a specific location, such as a nest or a food source, is crucial for many animals, including humans. This navigational task relies on integrating external environmental cues (allothetic cues), perceived through different sensory modalities, with self-generated information (idiothetic cues) about the animal’s movement and orientation. Path integration is a mechanism generally regarded as crucial for goal-directed navigation. This process is especially important for animals that need to repeatedly return to behaviourally relevant locations, such as their home or a profitable food source. Path integration allows animals to continuously track the distance and direction of their movements, enabling them to determine their current position relative to their starting point and to return directly to it. This strategy is fundamental to navigation, because it can be employed even in completely unfamiliar environments where environmental cues are unavailable for alternative navigational methods. However, path integration may also support more advanced navigation strategies by associating environmental features with direction and distance information in a place memory, contributing to map-like representations (McNaughton et al. 2006; Biegler 2010; Webb 2019).

Research has explored path integration across a wide range of species, from arthropods to vertebrates, including humans (Loomis 1999; Wehner and Srinivasan 2003; Etienne and Jeffery 2004; McNaughton et al. 2006; Wolf 2011; Heinze et al. 2018; Patel et al. 2022; Titova et al. 2023). Most studies have focused on terrestrial navigation, where locomotion is by walking and the number of steps taken by the animal provides a natural distance scale (Wittlinger et al. 2006, 2007). However, path integration may also contribute to navigation in three-dimensional environments, such as those encountered by flying animals or species in aquatic habitats. In addition to the inherent challenges of orientation in three-dimensional space without ground contact, the motion of the surrounding medium-such as wind or water currents-further complicates the estimation of distances travelled, a requirement for path integration (Aharon et al. 2017; Patel and Cronin 2020; Sibeaux et al. 2024).

**Fig. 1:**
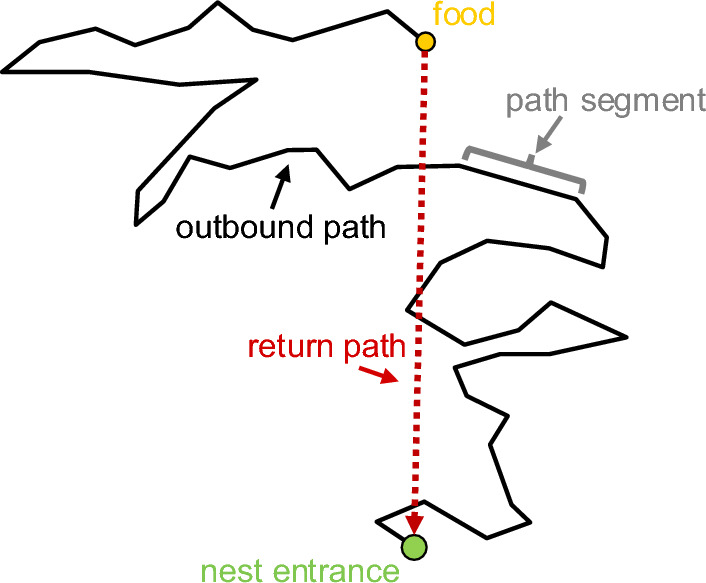
Schematic of the basic principles of path integration. An animal leaves its home in search of food. Since the food’s location is unknown, the animal takes an exploratory outbound path made up of multiple segments. Each segment has a specific length and direction, which can be represented as a vector. Path integration involves the animal continuously updating a vector that points back to its starting position, calculated by summing the vectors of its individual path segments. This enables the animal, upon finding food, to navigate directly back to its home using the final, computed goal vector

Path integration relies on four key components: (i) estimating the direction and (ii) the distance travelled for each path segment, (iii) combining direction and distance travelled into a movement vector, and (iv) integrating these segmental vectors into a final goal vector that represents the direct route back to the starting point (Fig. 1). To achieve this, animals must track their translational and angular displacements using various sensory mechanisms. Direction and distance information are assumed to be processed separately and then to be combined into a single vector. Directional information can be obtained from allothetic cues, such as the Earth’s magnetic field, the position of the sun (directly or via the celestial polarized light pattern), the moon and stars, or prominent landmarks and the horizon profile (Rossel and Wehner 1986; Dyer 1987; Wiltschko and Wiltschko 1990; Lohmann and Lohmann 1996; Dacke et al. 2003; Wehner and Müller 2006; Guerra and Reppert 2015; Seelig and Jayaraman 2015; Freas et al. 2017; Fleischmann et al. 2018; Foster et al. 2019; Wehner 2020; Haberkern et al. 2022; Warrant et al. 2024). Idiothetic cues, such as proprioceptive inputs from body rotation, may also play a role in determining directional information (Etienne and Jeffery 2004). The estimation of distance travelled can involve several mechanisms, such as somehow integrating the number of steps in walking animals, measuring energy consumption, or using odometry based on the optic flow generated on the eyes by the animal’s own movement (von Frisch 1967; Proffitt et al. 2003; Frenz and Lappe 2005; Wittlinger et al. 2006, 2007; Lappe and Frenz 2009; Walls and Layne 2009; Srinivasan 2014). These different sources of information can be used individually or in combination to optimize navigation.

Notwithstanding the broad relevance of path integration across species, we focus on insects, and specifically Hymenoptera, because they have been particularly well studied in this context. Among Hymenoptera, certain ant species serve as exceptional reference systems for path integration due to the extensive research on their navigation strategies, especially desert ants navigating environments where visual landmarks are scarce. In such settings, path integration is vital to locate the often barely visible nest entrances over long distances (Wehner 2020). Displacement experiments are a key method for analysing path integration: after following a winding path to locate a food source, ants—even after being spatially displaced from their initial location—reliably orient themselves in the original direction toward their nest and, upon reaching the approximate distance where they expect the nest to be, switch to search behaviour (Wehner 2020). Even in insects that typically move primarily by flying, displacement experiments have been successfully conducted under conditions in which the animals were experimentally confined to walking. These experiments have also yielded strong evidence for path integration (Bisetzky 1957; Patel et al. 2022; Titova et al. 2023). Ants mainly use a celestial compass (based on the sun’s position and the pattern of polarized skylight) for directional information and integrate the steps they take to estimate distance travelled (Wehner 2020). Research has now shown that both compass and distance information are encoded and linked within the central complex of the insect brain (Pfeiffer and Homberg 2014; Turner-Evans and Jayaraman 2016; Webb and Wystrach 2016; Heinze 2024). Several computational models inspired by these neural mechanisms further support conclusions drawn from experimental findings (Turner-Evans and Jayaraman 2016; Fiore et al. 2017; Stone et al. 2017; Heinze et al. 2018; Honkanen et al. 2019; Le Moel et al. 2019; Pisokas et al. 2020; Goulard et al. 2023; Heinze 2024).

Although the evidence for path integration is robust in ants, the situation is more complex in flying Hymenoptera, where this issue has been most thoroughly studied in honeybees. The challenges in studying path integration in bees are largely methodological. The flying mode of locomotion and their extensive foraging range make it difficult to experimentally isolate path integration from other sources of information used for goal-directed navigation. Although displacement experiments with bees have been carried out for quite some time (von Frisch 1967; De Marco and Menzel 2005; Riley et al. 2005), interpreting the results purely in terms of path integration is challenging, as the bees may have had access to additional environmental cues in their natural surroundings.

The directional cues used in walking, particularly the celestial compass, are also used in flight (von Frisch 1967; Rossel and Wehner 1982, 1986; Dovey et al. 2013). However, estimating distance travelled in flight introduces additional complexities. Potential mechanisms, such as integrating the number of wingbeats, measuring energy expenditure, or using mechanical sensors would require combining these measures with wind speed estimates-assuming a flying animal can measure wind speed (over ground) at all. Another possibility involves inertial navigation, which takes into account the animal’s internal measurements of movement (von Frisch 1967; Esch and Burns 1996). However, over the past 30 years, evidence has converged toward optic flow, which is generated on the eyes during self-motion in a static environment, as the primary source of information for estimating flight distance travelled (see Sect. Estimating travel distance using optic flow information). Yet, optic flow is inherently ambiguous because it depends on the ratio of the speed of locomotion to the distance of environmental structures in the visual field, providing only indirect information on distance travelled (see Sect. Optic flow and spatial information: ambiguities and predictions for flight distance estimation).

Despite these complexities, path integration in flight is often assumed to resemble that in walking insects. Although optic flow is widely recognized as the primary source of distance information in flight, its inherent ambiguities are frequently overlooked or insufficiently addressed. Therefore, this review aims to critically evaluate the experimental evidence for path integration in flying Hymenoptera and to identify gaps in our understanding. The evidence unequivocally supports the celestial compass as the most important source of directional information for bees (see above). Therefore, we focus on the still unresolved aspects of odometry—specifically, the determination of flight distance. After summarising evidence that bees indeed utilize both compass and distance information for navigation, though not necessarily performing genuine path integration, we address the following questions:

- What are the challenges of using optic flow to estimate flight distance? What predictions can be made about behavioural performance on this basis?
- What evidence supports optic flow playing the primary role in estimating flight distance? Are the predictions based on optic flow properties met?
- Are flight distances estimated during the outbound flight from the nest to a food source, during the inbound flight when returning to the nest, or during both?
- Since optic flow may significantly vary across different parts of the visual field depending on the spatial layout to the environment, which eye regions are responsible for estimating flight distance?
- Are the measured directions and lengths of segments of a flight path effectively linked to form a vector, as required for path integration?

Eventually, we will argue that the evidence for genuine path integration in flying insects is currently weak and that further research is definitely needed (Table 1). However, from an ecological perspective, we argue that reliable path integration may be far less important for flying insects, which usually navigate in visually well-structured environments, than it is for desert ants in an environment that offers few options other than path integration for finding the way to the nest (see Sect. Conclusions: Path integration-is it a ubiquitous ability in Hymenoptera?).

**Table 1:** Summary table of studies on different aspects of flight distance estimation by bees

Research topic	Species /experimental setting	Indicator of flight distance	Key results	Publication
Relationship flight distance / WD duration	Honeybee / outdoors: feeders placed at different distances from hive	WD	Linear relationship between WD duration and flight distance • Depends on environment • Interindividual variation > environmental variation	von Frisch and Jander 1957; Wenner 1962; Esch et al. 2001; Schürch et al. 2013; Schürch et al. 2019
Estimating travel distance by optic flow information	Honeybee / outdoors setting; goal attached to balloon at different heights	WD	WD duration increases with amount of OF	Esch and Burns 1995
	Honeybee / outdoors setting in combination with textured flight tunnels inducing strong OF	WD	WD duration increases with amount of OF	Srinivasan et al. 2000; Esch et al. 2001; De Marco and Menzel 2005
	Honeybee / outdoors in differently structured environments	WD	WD duration increases with amount of OF	Tautz et al. 2004
Where is distance traveled estimated? Outbound vs. inbound flight	Honeybee / outdoors; displacement of feeder and/or feeder with bee over variable distances	WD	Partly contradictory observations between studies with respect to significance of outbound and return flight. Overall: evidence that in addition to the outbound flights, also the inbound flights play a role in determining flight distance signaled by the WD	Heran and Wanke 1952; Heran 1956; Otto 1959; von Frisch 1967
	Stingless bee / flight tunnels with wall textures inducing OF of variable strength in different eye regions	SL	Learning of flight distance: primarily on return flight, but to some extent, also on outbound flight	Hrncir et al. 2003
	Honeybee / flight tunnels with wall textures inducing OF of variable strength in different eye regions	SL	Learning of flight distance: primarily on outbound flight	Srinivasan et al. 1997; Srinivasan et al. 1998
	Honeybee / outdoors & flight tunnel	WD	Learning of flight distance: primarily on outbound flight	De Marco and Menzel 2005
	Honeybee / outdoors; displacement to unknown location after foraging trip to food source	WD	Learning of goal direction on return flight, because it was signaled by WD after first return flight	Edrich 2015
Eye regions and OF integration for estimating flight distance	Stingless bee / flight tunnels with varying wall textures	SL	Lateral and ventral visual field	Hrncir et al. 2003
	Honeybee / light tunnels with varying wall textures	SL	Predominantly lateral visual field	Srinivasan et al. 1997
Are distance and direction estimates combined into one vector?	Honeybee / outdoors	WD	WD signals direction of shortcut, but actual distance traveled (i.e. not shortcut distance): PI vector not represented	von Frisch 1967
	Honeybee / outdoors & flight tunnel mimicking a triangle completion task	WD	Inexperienced bees signal absolute subjective distance flown (not shortcut), but not virtual direction of goal Experienced bees signal absolute subjective distance flown (not shortcut) and approx. virtual direction PI vector not represented-neither by inexperienced nor by experienced bees	De Marco and Menzel 2005
	Honeybee / flight tunnels with differently aligned above polarization patterns	WD	WD signals direction of shortcut to goal, but absolute subjective distance traveled PI vector not represented	Evangelista et al. 2014
	Honeybee / flight tunnels with variable amount of access to above polarization pattern	WD / SL	**WD**: signals distance traveled based on OF on all path sections regardless of polarization availability. **SL**: estimation of distance travelled takes into account only path sections on which polarization information was available	Dacke and Srinivasan 2008
Role of experience in estimating flight distance	Honeybee / outdoors; distance of food source was varied in different phases of experiment and consequences for WD analysed	WD / SL	**WD**: relies on gradual incorporation of previously learned spatial information, **SL**: bee’s own foraging hypothesized to be more influenced by current distance information	Chatterjee et al. 2019
	Honeybee / outdoors and flight tunnels; cue conflict between OF induced by flight tunnel and environmental structures seen in the upper visual field	WD	If bee is unfamiliar with the environment, tunnel-based OF is used for distance estimation; if familiar with environment, distance information provided by the tunnel-based OF is not used	De Marco and Menzel 2005; Menzel and Galizia 2024

*WD* waggle dance, *SL* search location, *OF* optic flow

## Bees utilize distance and compass information for navigation

**Fig. 2:**
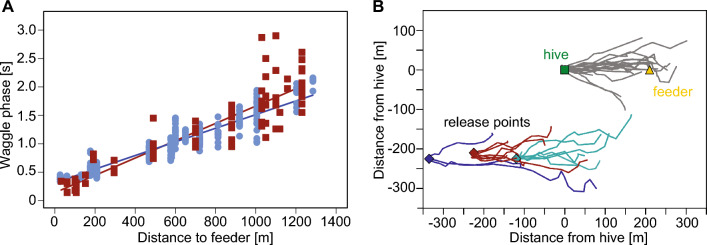
Flight distance information conveyed by waggle dance. **A **Increase in the duration of the waggle phase of the dance as a function of the distance to a food source. Each data point represents an individual dance of a marked bee that was trained on a food source located at a certain distance from the nest. The data were obtained in two studies and in different environments (red squares: Wenner 1962; blue dots: Schürch 2013); the respective regression lines are shown in the corresponding colour (modified from Schürch 2013). **B **Displacement experiment providing evidence that the waggle dance conveys information about direction and distance to a goal. Flight paths of bees that left the hive at position (0,0) (green square) or were displaced to three other release points by up to more than 300 m (coloured diamonds) that were out of sight from the hive, after following a waggle dance of other bees to a food source (orange triangle). As a reference, individual flight paths from the nest are shown in grey. Bees that were displaced to locations southwest of the hive before their departure are shown in different colours corresponding to the point of release. Most bees flew in directions and distances that varied around the direction and distance between the food source and the hive, regardless of whether or where they were released. This indicates that the waggle dance on average represents the respective goal vector (modified from Riley et al. 2005)

The ability of flying Hymenoptera, particularly bees, to estimate their flight direction and distance has long been demonstrated in a variety of ways. A significant body of evidence highlights the importance of the honeybee waggle dance, a complex, multi-component signal that conveys key information about food sources, particularly their location and value to the colony (von Frisch 1967; Seeley 1995; Michelsen 2003; Grüter and Farina 2009; I’Anson Price and Grüter 2015; Palmer et al. 2024). Specifically, the dance reflects the direction of a path relative to the sun and/or the polarization pattern in the sky and is interpreted by the bees to be recruited based on its orientation relative to the vertical axis within the hive (von Frisch 1967; Dyer 2002). Several studies have shown by training bees to visit feeders placed at different distances from the hive that the duration of the waggle phase of the dance conveys information about the distance between the hive and the food source. These studies show that there is an approximately linear relationship between distance to the food source and duration of the waggle phase for flight distances from relatively close to the hive (< 100 m) to distances over 1 km, with the slope of this relationship varying between different environments (Fig. 2A) (von Frisch and Jander 1957; Wenner 1962; Esch et al. 2001; Schürch et al. 2013, 2019). However, the variability in waggle phase duration between different bees, even in a given environment, was shown to be greater than the variability in the relationship between foraging distance and waggle phase duration in different environments. Thus, inter-individual variability between bees in natural landscapes seems to mask to some extent the dependence of bee distance measurements on specific environmental characteristics and underlines that distance information is only signalled with limited reliability by the waggle dance (Schürch et al. 2019).

Targeted experimental manipulations of bee behaviour, combined with advanced radar tracking of flight trajectories in natural foraging environments, have greatly enhanced our understanding of bee navigation. To determine the specific information that bees recruited by the waggle dance can decode, it was essential to test them in an unfamiliar environment devoid of previously learned cues. This can be achieved by displacing the bees to an unknown location before they leave the hive. If the only information available to the bees is the direction and distance to the food source as encoded in the waggle dance, they should fly along this vector towards a virtual food source, regardless of their displacement. Indeed, observations show that bees generally follow a relatively straight path towards the direction and distance they would have travelled from the hive to the food source, even after displacement. If the goal is not found, they then perform search flights around the anticipated goal location (Riley et al. 2005; Wang et al. 2023) (Fig. 2B).

Although the waggle dance signals the approximate direction and length of a learned goal vector, it provides no information about how the dancing foragers acquired this information, particularly whether it was derived from path integration during a single outbound flight. Alternatively, the dancing bee may have acquired the direction and distance information during the inbound flight or through repeated trips between the food source and the hive, relying on accumulated experience and additional sources of information.

## Optic flow and spatial information: ambiguities and predictions for flight distance estimation

Optic flow, defined as the retinal motion of the environmental image generated dynamically on the eyes during self-movement, is considered the most plausible information source for estimating flight distances in bees (see Sect. Estimating travel distance using optic flow information for evidence). However, the relationship between optic flow and the distance flown is intricate, making it challenging to directly translate one into the other. Therefore, we review the relevant aspects of optic flow and its neural representation to predict how bees may estimate flight distance.

Moving through cluttered environments generates retinal image flow. Geometrically, the strength of the translational component of optic flow at a given flight speed depends on the three-dimensional structure of the environment: the closer the animal is to objects or the ground, the higher the retinal velocities in the corresponding part of the visual field. More precisely, optic flow at a given flight speed is proportional to the nearness of objects. Hence, the translational component of optic flow is ambiguous because it depends on both the distance to objects in the environment and the animal’s speed (Longuet-Higgins and Prazdny 1980; Koenderink 1986; Heeger and Jepson 1992; Egelhaaf 2023).

Note that the meaning of “distance”—referring to the *distance of the animal to objects in the environment*—must be strictly distinguished from its common usage in the context of path integration, where it refers to the *distance travelled by the animal*. To avoid any misunderstandings, we will use “nearness”—the inverse of the distance of the animal to objects in the environment $\:n=\raisebox{1ex}{1}\!\left/\:\!\raisebox{-1ex}{d}\right.$—when referring to the distance to objects at a given position along the bee’s path, and “distance” when we mean the distance travelled.

If the distances travelled cannot be measured directly using a distance scale, as is done in walking animals such as ants by somehow multiplying representations of their step length by the number of steps (Wittlinger et al. 2006, 2007), it can alternatively be determined formally by calculating the product of flight speed $\:v$ and flight time $t:s = v \cdot t$ (assuming a path segment flown at constant speed). However, optic flow cannot be used as a direct proxy for flight speed as a basis for estimating flight distance, because it is also influenced by the 3D layout of the environment, making it ambiguous. Thus, for a given flight speed $\:v$, optic flow varies systematically with flight altitude $\:h$, affecting retinal velocities $\:{v}_{r}$ in the ventral visual field $\left( {v_{r} \propto \frac{v}{h}} \right)$, and with the nearness $\:n$ of obstacles, influencing retinal velocities measured in the lateral viewing direction: $v_{r} \propto v \cdot n$.

This conclusion stems entirely from the geometry of optic flow generation and is independent of the specific mechanisms used to detect or compute it. Even with a hypothetical perfect retinal velocity sensor, the animal can only measure the product of flight speed and nearness as a single combined value. This makes optic flow an inherently ambiguous information source for disentangling these two parameters. In the discussion that follows, we primarily address this geometric ambiguity in the optic flow signal, rather than any additional ambiguities introduced by the specific peculiarities of biological motion detectors.

**Fig. 3:**
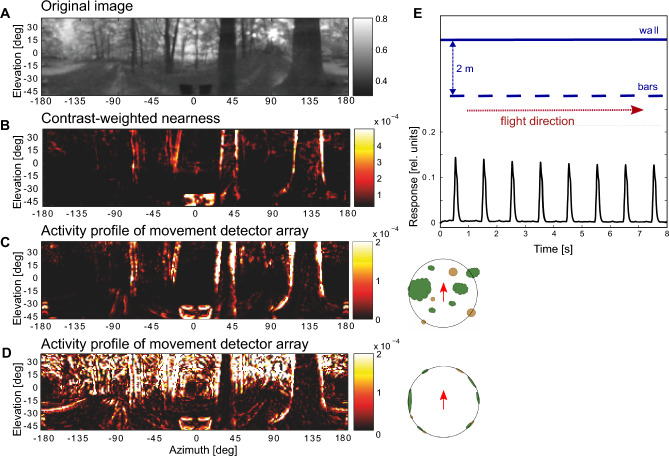
Optic flow and spatial information: Ambiguities and predictions for flight distance estimation: Activity profile of a retinotopic array of local movement detectors represents contrast-weighted nearness in response to the optic flow induced by translatory movement in natural environments. **A–D **All images represent one instant of time during a longer translation sequence. **A **Original input image of a natural scenery (tone-mapped HDR image). **B **Contrast-weighted nearness map: The nearness is multiplied by the local contrast at the corresponding image location, resulting in the largest contrast-weighted nearness values at the edges of nearby objects (colour code in arbitrary units). **C **Activity profile of the array of movement detectors while the observer moves through the environment with its natural depth structure (see inset at right). The activity profile is given by the absolute values of horizontally and vertically aligned local movement detectors; model simulations based on an elaborated version of the correlation-type movement detector (colour code arbitrary units). The activity profile of the movement detector array reflects the contrast weighted nearness structure of the three-dimensional environment. **D** Activity profile of the array of movement detectors at one instant of translatory motion after the depth structure of the environment has been equalized by projecting it on the surface of a sphere (see inset at right). Model simulation as in C. The activity profile now reflects all contours in the environment irrespective of their distance (data from Schwegmann et al. 2014a, 2014b). **E **Optic flow and, accordingly, local motion detector responses are temporally modulated during translation in an environment with depth structure. Top: Spatial layout of the artificial 3D environment and the flight trajectory (red arrow) at a distance of 0.5 m parallel to a row of bars and a wall 2 m behind the bars (dark blue). Bottom: Time course of absolute response values of horizontally and vertically aligned local movement detectors averaged across the vertical extent of the array at azimuth 90° (sideways view); model simulations are based on an elaborated version of the correlation-type movement detector with adaptive properties. The response amplitude reflecting the optic flow fluctuates when passing the nearby bars, although the translation velocity is constant (data from Li et al. 2017). Thus, in cluttered environments, optic flow is an unreliable indicator of the animal’s actual speed

The specific dependence of optic flow on the nearness of environmental structures has been analysed for different functional contexts in artificial and natural environments based either on the geometrically correct optic flow (Ravi et al. 2022) or on experimentally based models of motion detection in insects (Eckert and Zeil 2001; Zanker and Zeil 2005; Schwegmann et al. 2014a). These models effectively describe the fundamental computations involved in the insect visual motion pathway (Borst and Egelhaaf 1993; Egelhaaf and Borst 1993; Borst et al. 2010; Egelhaaf et al. 2014; Mauss and Borst 2019). Because the retinal velocity of an object scales with nearness during translational locomotion, a nearby object will elicit stronger local motion detector responses than more distant ones. This effect is so strong that it can be exploited to efficiently segment a visual scene into near and far objects during translatory movement in a natural environment (Zanker and Zeil 2005; Egelhaaf et al. 2014; Schwegmann et al. 2014a; Ullrich et al. 2015; Li et al. 2017; Ravi et al. 2022). Furthermore, the array of local motion detectors mostly represents the nearness of object contours rather than the nearness of the objects’ entire surfaces because motion detector responses are induced only by textured surfaces or object boundaries. This feature is illustrated in Fig. 3A–D, which depicts the model simulations of an array of motion detectors responding to constant-velocity translational movement in a cluttered natural environment at a specific moment in time (Fig. 3B, D). However, regardless of whether optic flow is represented by realistic motion detectors or abstractly through its geometric properties, its representation across the visual field is non-uniform and exhibits spatial discontinuities. Although, this is essential for spatial vision in dense environments, such as collision avoidance (Bertrand et al. 2015; Lecoeur et al. 2019; Ravi et al. 2022), it has undesirable consequences when calculating flight speed from the motion pattern on the retina, since then different areas of the visual field may indicate different flight speeds even if the actual flight speed is constant. An approach to reduce these ambiguities and discontinuities is to spatially average the motion detection output over appropriate areas of the visual field (Meyer et al. 2011; Lecoeur et al. 2019), thereby improving the estimate of flight speed. However, even with this averaging, the estimate remains dependent on the mean distance of objects within the respective area of the visual field.

The dependence of the optic flow on the nearness of objects has not only spatial but also temporal consequences with regard to determining the flight distance. When an animal is moving in a cluttered environment, the objects moving across the retina cause significant temporal modulations in motion detector responses at a given retinal position, because the nearness of the surrounding objects is continually changing, even if the animal is traveling at a constant speed. This feature is illustrated in Fig. 3E, which presents a model simulation of translational motion along a row of nearby textured objects positioned in front of a similarly textured wall. The strength of the time-dependent response modulations increases with increasing distance between the objects and the wall (Li et al. 2017). If the optic flow is used as a proxy for flight speed, these modulations might indicate that flight speed is continually changing, although it is kept constant all the time. These temporal modulations, thus, pose a major challenge for estimating flight distance and, consequently, for optic flow-based path integration.

At what spatial scale can optic flow be effectively utilized for estimating distance travelled, considering that retinal velocities decrease and, thus, the corresponding motion signals rapidly decrease with decreasing nearness of environmental structures? This question is pertinent because the performance of visual mechanisms, especially in biological systems, is constrained by noise present at all levels of the visual pathway. Systematic electrophysiological experiments on output neurons of the visual motion pathway in flies revealed that at a translational speed of approximately 0.5 m/s within a flight arena, the optic flow-induced neuronal responses reliably conveyed spatial information only up to a range of about 1 m; beyond this distance, the motion signals were too weak and fell below the noise level (Kern et al. 2005). Hence, for determining flight distance based on optic flow accurate measurement is feasible only if the animal flies sufficiently close to environmental objects or the ground. Since the strength of optic flow is proportional to flight speed, the spatial range of environmental structures that generate detectable optic flow—and can thus be used for distance estimation—scales linearly with flight speed. Extrapolated to cruising flight speeds of bees in natural environments, which are estimated to vary between around 4–8 m/s (Heran 1956; Wenner 1963; Osborne et al. 1996; Riley et al. 2005), detectable optic flow is generated when objects or the ground are closer than approximately 10–14 m. Although these estimates are approximate, it is evident that, as a consequence of the limited reliability of sensory mechanisms in biological systems, spatial structures must be sufficiently close to produce detectable optic flow, which then allows to estimate flight speed and, subsequently, flight distance.

**Fig. 4:**
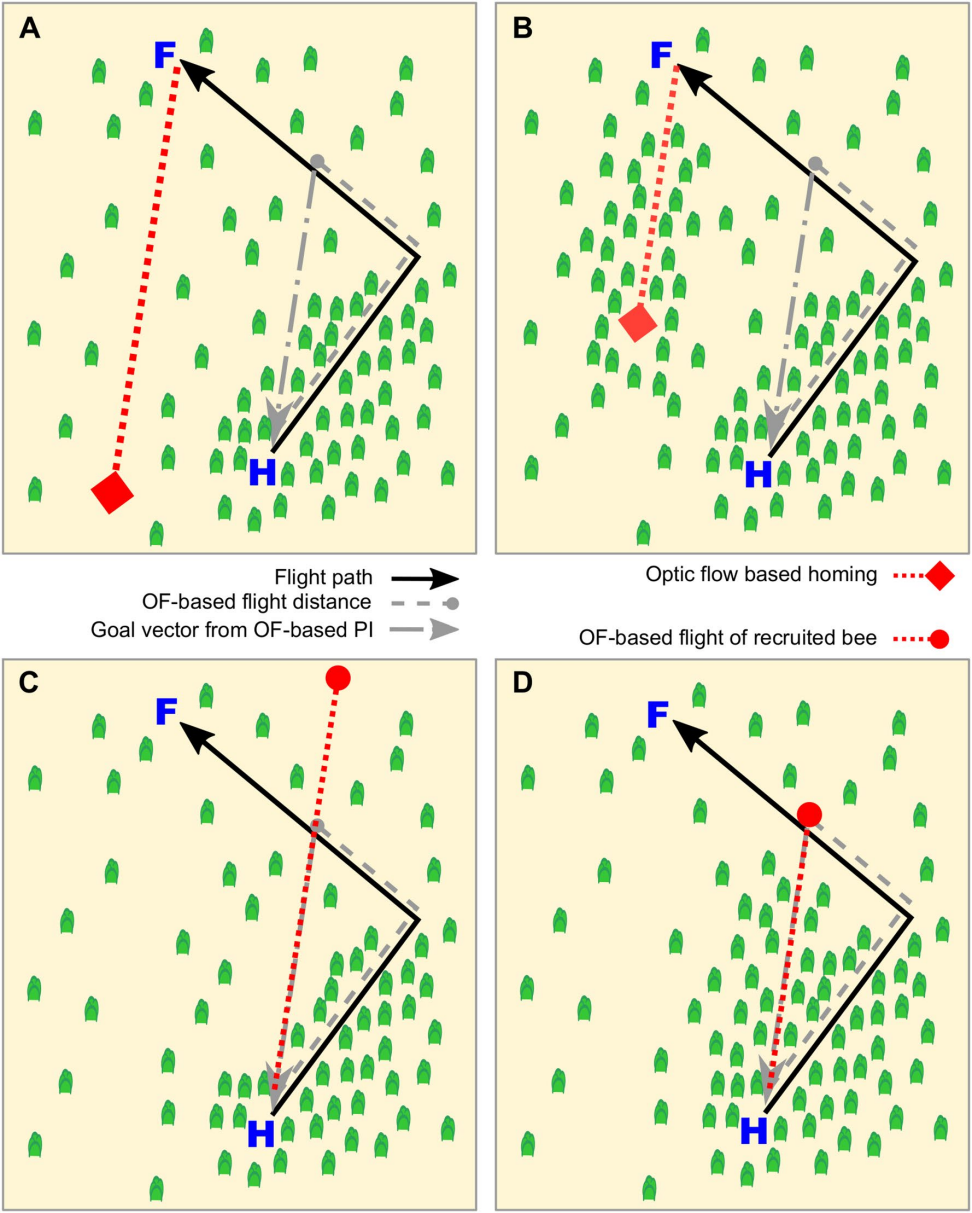
A schematic illustrating the potential consequences of optic flow-based path integration using information from the entire visual field in environments with varying levels of clutter, focusing on its impact on homing bees and those recruited via the waggle dance vector. Path integration is illustrated for a bee flying from its nest (blue H) to a food source (blue F) along two sides of a right-angled triangle (black kinked arrow). The first leg traverses densely structured terrain (green objects), generating strong optic flow, while the second leg crosses open terrain with weaker optic flow. Consequently, the bee underestimates the distance of the second leg relative to the first (dashed grey lines). Thecalculated goal vector (dashed-dotted grey arrow) guides the return flight (red dotted lines), which is terrain-dependent: **A** In loosely structured environments, the bee flies farther to reach the presumed goal (red diamond). **B** In dense terrain, the same optic flow is achieved over shorter distances. In both cases, the nest is not reached. For recruited bees, the optic flow-based goal vector communicated via the waggle dance similarly influences their flight. **C** In open terrain, the food source appears farther away (red circle). **D** In dense terrain, the food source appears closer. In all scenarios, the actual food source (blue F) is not reached, as flight distances through open terrain are consistently underestimated compared to denser terrain. These errors are independent of the calibration method for translating optic flow into flight distance and highlight the consistent impact of terrain-induced optic flow discrepancies on navigation
- *Temporal fluctuations*: In cluttered environments, such as when flying through vegetation, temporal modulations of optic flow make it difficult for instantaneous motion signals to accurately reflect flight speed and, consequently, flight distance. This issue is particularly pronounced over short time scales and relatively short flight distances, as temporal fluctuations can only be reduced through temporal integration over brief periods. This limitation arises especially in cluttered environments because a constant direction and speed can only be maintained for a short duration in such environments (see Fig. 5). Consequently, relying on optic flow for flight distance estimation and path integration on a small spatial scale is highly challenging. This limitation is particularly important when interpreting experimental studies, especially those involving flying insects in small-scale laboratory settings. To our knowledge, this issue has not been explicitly addressed in prior research, underscoring the need for systematic experimental and modelling analyses to explore it further.

Based on the spatial and temporal characteristics of optic flow and its presumable representation in the insect visual motion pathway, several predictions can be made about the performance of animals using optic flow-based estimates of distance travelled:

- *Environmental dependence*: Since optic flow-based flight speed estimates depend on the 3D layout of the environment, the resulting distance estimates are expected to vary based on the specific environments traversed, particularly if information from the lateral visual field is incorporated. This introduces significant challenges for path integration if the environments encountered during outbound and return flights differ substantially in their spatial characteristics (see Fig. 4). Even if the animal primarily relies on optic flow in the ventral visual field to estimate its speed and, consequently, its flight distance, the optic flow is affected by variations in ground structure, even at a constant flight altitude - for example, when flying over bushes or other elevations. Moreover, even over completely flat terrain, optic flow remains inherently ambiguous, as it depends on both the distance to the ground and the flight speed above it.

**Fig. 5:**
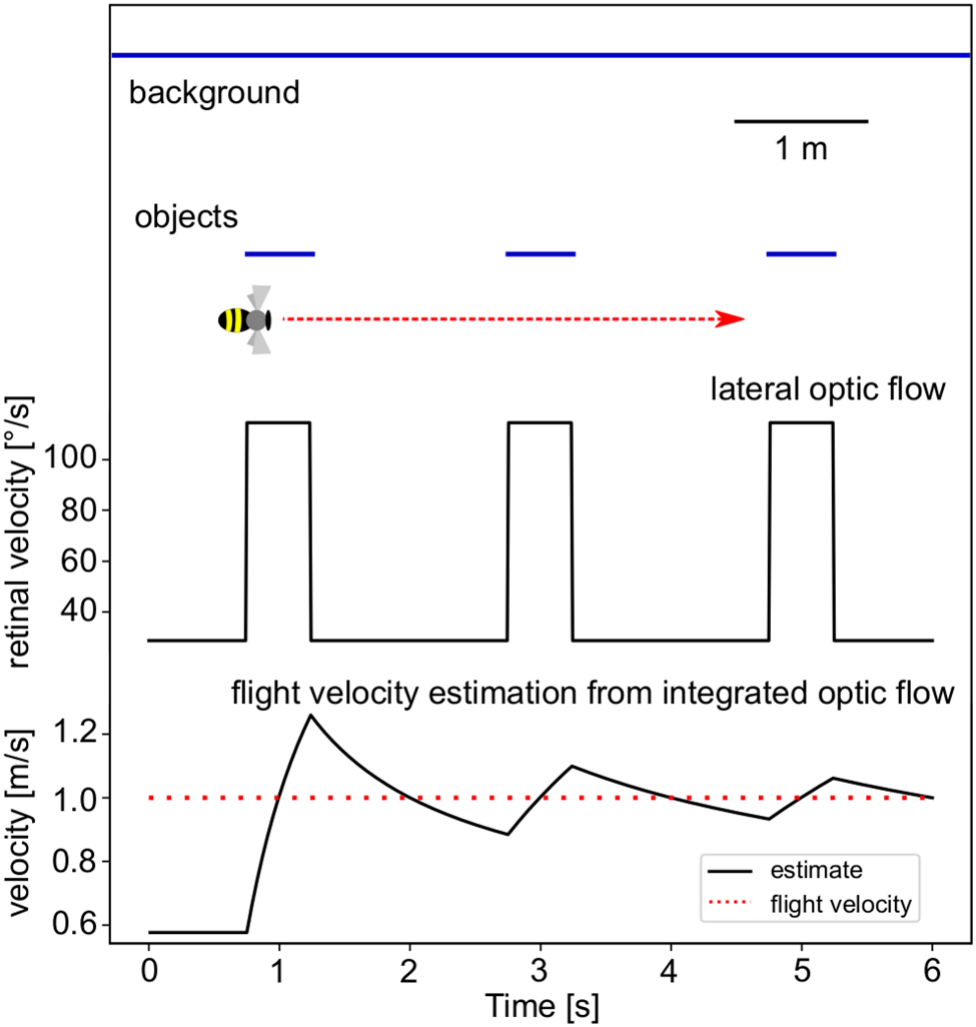
Consequences of optic flow integration for flight speed representation in a cluttered environment. Top panel: Spatial layout of an artificial 3D environment, showing the flight trajectory (red arrow) at a distance of 0.5 m, parallel to a row of textured bars with a textured background wall positioned 2 m behind the bars. Middle panel: Retinal velocity in the lateral visual field at 90° from the flight direction. Despite a constant flight velocity, the retinal velocity shows significant temporal modulation (velocity steps when the bee passes the objects). Bottom panel: Progressive attenuation of these fluctuations through temporal averaging, reducing errors in flight velocity estimation. This averaging is only meaningful if the animal maintains constant speed and direction - conditions typically sustained for only a few hundred milliseconds during free flight, particularly in cluttered environments

These predictions are based on the assumption that the estimation of flight speed and, subsequently, flight distance relies exclusively on the translational component of the optic flow, *without incorporating additional information*. However, if additional information, such as flight altitude, were available from other sources and the optic flow scaled accordingly, a more accurate estimate of flight speed and distance could be derived from the optic flow in the ventral visual field. If the animal were also to manoeuvre orthogonally to its primary direction of motion, it would theoretically be possible to extract nearness information from optic flow. The orthogonal movement introduces acceleration components into the optic flow, allowing the estimation of the nearness of surrounding structures, which could then be used to scale the translational optic flow, providing a flight speed approximation that is less influenced by the spatial structure of the environment (Bergantin et al. 2021). However, there is no evidence yet that insects use such a calibration mechanism (see Sect. Estimating travel distance using optic flow information), even though they adaptively use the optic flow to control their flight speed and altitude, either by adjusting it to a setpoint depending on the nearness of environmental objects (Srinivasan et al. 1996; Fry et al. 2009; Baird et al. 2010; Portelli et al. 2011; Baird and Dacke 2012; Kern et al. 2012; Linander et al. 2015, 2016; Serres and Ruffier 2017), or by changing their altitude according to the profile of the ground (Baird et al. 2006; Portelli et al. 2010) including crashing with the ground if it does not induce any optic flow (Serres et al. 2022). It is important to note, however, that these speed and altitude control mechanisms do not result in a correct estimate of flight speed, as would be required in the context of path integration as a basis for determining the distance travelled. All these investigations were conducted under carefully controlled laboratory conditions, allowing precise manipulation of the environment to identify causal relationships. While it would be highly beneficial to systematically study the flight paths of bees in natural conditions - particularly the regulation of their altitude - this remains methodologically challenging despite significant advancements in technology, such as radar tracking and drones for following bees (Lihoreau et al. 2012; Woodgate et al. 2016, 2021; Vo-Doan et al. 2024).

## Estimating travel distance using optic flow information

**Fig. 6:**
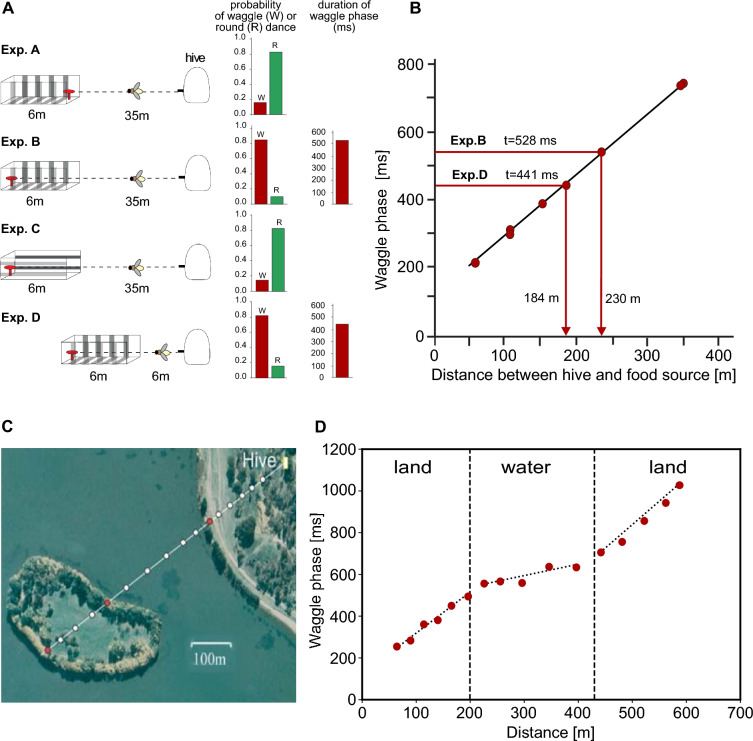
Estimating travel distance using optic flow information. **A, B **Behavioural analysis of how honeybees estimate the distance between their hive and a food source and communicate this information to other foragers via their waggle dance. **A **Left: Experimental layout using flight tunnels. Right: Probabilities with which a round dance signalling short flight distances (R; green bars) or a waggle dance (W; red bars) was performed by the bees in the respective situations and the duration of the waggle phase of the dance, with which the distance between food source and nest perceived by the bee is indicated. The walls of the tunnel were either covered with a texture that contained vertically oriented stripes inducing strong optic flow (Exp. A, Exp. B, Exp. D) or horizontally aligned stripes inducing only weak optic flow (Exp. C). The bees were trained to collect sugar water from a food source (indicated by red object). When the food source was placed at the entrance of the tunnel (Exp. A), the bees performed mainly round dances after returning to their hive, signalling a short distance to the food source. When the food source was placed at the end of the tunnel containing vertically oriented texture (Exp. B), the returning bees performed mainly waggle dances, signalling much larger distances to their nestmates, although the actual travel distance was not much larger. A food source at the same distance, however, located in a tunnel with horizontally oriented stripes (Exp. C), led mainly to round dances. Even when the tunnel covered with vertical contours and the food source close to its end was placed near to the hive (Exp. D) mainly waggle dances were performed, with, the waggle phases being shorter than those in Exp. B. These experiments suggest that flight distance is measured in terms of optic flow. **B **Calibration of the odometer of the bee. Mean duration of waggle dances elicited by outdoor feeders at various distances to the hive. Also shown are the mean durations of waggle dances measured in Exp. B and Exp. D and their equivalent outdoor flight distances, as read from the regression line. These findings show that optic flow-based distance measurements depend much on the spatial layout of the environment (adapted from Srinivasan et al. 2000). **C, D**. Increase of waggle phase duration with feeder distance depends on strength of optic flow induced in differently structured natural environments. **C **The bees were trained to fly from the hive initially over land, then over water, and finally again across land. The white dots depict successive locations of the feeding station (in the water they were placed on a boat that was kept stationary at different locations). The red dots along the route depict the shoreline stations, which represent the boundaries between land and water. **D **Mean waggle phase duration increases much more with feeder distance for flight sections over land than for flight sections over water most likely because the land sections lead to a much stronger optic flow than the less structured water (modified from Tautz et al. 2004)

According to current knowledge, the optic flow generated on the eyes during flight is considered the most plausible source of information for estimating distance travelled by flying bees. A substantial body of evidence supports this view. One of the most compelling lines of evidence comes from experiments where bees were trained to forage in flight tunnels with varying configurations, allowing for a systematic manipulation of optic flow through changes in tunnel width and wall texture. In these experiments, when large optic flow was induced by tunnel textures, bees signalled much greater flight distances through their waggle dance compared to waggle phase duration as had been calibrated in natural environments with food sources placed at various distances from the hive. Conversely, when the tunnel walls were covered with patterns that induced minimal optic flow, the distances indicated in the waggle dance were much smaller (Fig. 6A, B) (Srinivasan et al. 2000). This and several other studies (Esch and Burns 1996; Esch et al. 2001; De Marco and Menzel 2005) suggest that bees use information about optic flow to estimate their flight distance (Table 1).

What do these results, obtained under controlled laboratory conditions on optic flow-based distance estimation mean for corresponding estimates in natural conditions? The relationship between waggle phase duration and the distance of a food source from the nest varies across different environments. However, interpreting these variations in terms of the spatial arrangement of the environment and the resulting optic flow is challenging due to the lack of detailed information about the specific spatial characteristics of these environments. Moreover, it is unclear what prior experiences the bees have had with the environment and what additional sources of information they thus may have learned to use alongside optic flow (see Sect. Bees utilize distance and compass information for navigation). Despite these complexities, two studies have provided plausible arguments that optic flow is also the essential basis for estimating flight distances in natural environments. In one study, bees were trained to learn the location of food sources attached to balloons placed at different heights above ground. The flight distance signalled by the waggle dance systematically decreased as the height of the food source increased-reducing the optic flow caused by the reduced nearness of the ground (Esch and Burns 1995). In another outdoor study, waggle phase duration was measured for food sources placed at increasing distances along a route with varying optic flow due to differences in surface texture. When bees were trained and tested to visit feeders along a route that first traversed land with a textured surface, then over water with minimal surface texture, and finally back over land, their waggle phase duration increased with a significantly flatter slope per increment in travel distance during the over-water flight segments compared to the over-land segments. This suggests that the different surface textures between the land and water segments, and therefore the different levels of optic flow, influenced how the bees encoded distance in their waggle dance (Fig. 6C, D) (Tautz et al. 2004). Note that in contrast to the flight tunnel experiments (Fig. 6A), where the spatially close tunnel walls induced a strong optic flow, the objects in the natural environment of this experiment were so far away from the animal, at least during long flight phases and especially over water (Fig. 6C), that they could not have induced a measurable optic flow (see Sect. Optic flow and spatial information: ambiguities and predictions for flight distance estimation). Therefore, optic flow in the ventral visual field probably played the dominant role for the flight distance signalled in the waggle dance. This conclusion mirrors the relative roles of lateral and ventral optic flow in controlling flight speed observed in flight tunnel experiments with varying widths. In narrow spaces, bumblebees primarily rely on lateral visual field information, whereas in sufficiently wider tunnels, they predominantly rely on optic flow from the ground (Linander et al. 2017).

The environment a bee navigates, and its spatial characteristics can differ significantly between the animal’s left and right visual fields, leading to distinct optic flow patterns experienced by each eye. To investigate the impact of asymmetric optic flow during learning a food source and its effects on subsequent foraging behaviour in a different asymmetric environment, experiments were conducted in controlled flight tunnel settings. The bees were shown to estimate flight distance nearly as accurately when optic flow is presented to only one eye as when both eyes receive the same information. Furthermore, information acquired monocularly remains useful even when perceived during the test phase by the eye that was minimally exposed to optic flow during the learning phase. This capability is functionally significant in highly asymmetric environments, as it allows the bee to navigate the same distance while dealing with the spatial asymmetries encountered in opposite flight directions, such as during outbound and inbound flights (Srinivasan et al. 1998).

These results emphasize that optic flow serves as the primary source for flight distance. However, in environments where objects are unevenly distributed, the optic flow—and consequently the estimated flight distance—can fluctuate significantly over time and across different regions of the visual field, depending on the specific local spatial characteristics (see Sect. Optic flow and spatial information: ambiguities and predictions for flight distance estimation and above). This variability can be mitigated by averaging signal modulations over longer flight distances (see Fig. 5) and employing integrative processes that combine inputs from various parts of the visual field (see above). Despite these mechanisms, a fundamental challenge remains: Determining flight speed and distance *via* optic flow is significantly influenced by the spatial layout of the environment.

It is important to note that bees are generally assumed to associate the goal vector they form-based on directional and route information-with perceived landmark constellations as they gain experience in a specific environment (Chittka et al. 1995; Vladusich et al. 2005; Menzel et al. 2010). These landmarks may facilitate accurate goal-directed flight when necessary. Recent research (Menzel and Galizia 2024), on the role of optic flow in flight distance estimation revealed that honeybees rely heavily on their experience with the environmental context when communicating flight distance through the waggle dance. When exposed to both the wall textures of a tunnel, which generate strong optic flow, and structures of a more distant natural environment, the latter significantly influenced the flight distance communicated by the waggle dance-provided the bees had prior experience with that natural environment. In the absence of such familiarity, the flight distance indicated by the waggle dance was based on the current optic flow, predominantly influenced by the flight tunnel. This suggests that when bees encounter a combination of tunnel-induced optic flow and familiar environmental cues, a conflict arises: the optic flow induced by the tunnel walls signals a longer flight distance, while the familiar environmental information suggests a shorter distance. In such cases, the previously learned environmental cues appear to take precedence, dominating the distance information conveyed by the waggle dance (Menzel and Galizia 2024).

## Outbound vs. inbound flight: when is distance travelled estimated?

In principle, the distance travelled could be estimated and learned based on either the outbound route—from the nest to the food source—or the inbound return journey. Alternatively, it is possible to combine information from both routes and across repeated visits to the same food source. Each approach has its own functional advantages and disadvantages.

*Estimating and learning the distance travelled on the outbound flight* is crucial for determining a home vector through path integration. The nervous system must vectorially integrate the different segments of the outbound journey to determine both the distance and direction needed for a direct return to the hive (Fig. 1). For a naive forager with limited environmental information, accurately estimating the outbound flight distance and associated directions may be essential for a successful return. Conversely, *estimating and learning the distance on the return flight* could enhance the reliability of the information conveyed through the waggle dance. By calculating distance on the more direct return trip, integration errors are minimized due to the shorter duration and less complex path compared to the outbound journey. Furthermore, when the returning bee flies and signals through its waggle dance the direct route to the hive that the recruited forager bee should then fly in the opposite direction, the ambiguities of the optic flow depending on the 3D structure of the environment should be greatly reduced, as the environment is largely the same for both the inbound and outbound flights.

The question of whether bees learn the distance flown on their way to a food source or on their return journey has been a topic of investigation already in early studies of foraging and homing behaviour in outdoors environments, with the waggle dance serving as a key indicator of orientation performance. These studies led to somewhat conflicting observations regarding the relevance of outbound and return flights. Overall, the results can be summarised that, although flight distance is primarily measured on the outward journey to the food source, bees also use distance information obtained on their return flight to the hive (Heran and Wanke 1952; Heran 1956; Otto 1959; von Frisch 1967). This issue was then systematically investigated by deliberately manipulating the environment between the bees’ learning and testing phases (Srinivasan et al. 1997, 1998; Hrncir et al. 2003; De Marco and Menzel 2005). These manipulations aimed to investigate the causal relationships between the environment and the bees’ perception of flight distance. In these studies, the bees’ search location in test situations or the duration of the waggle phase were used as indicators of the learned flight distance (Table 1).

**Fig. 7:**
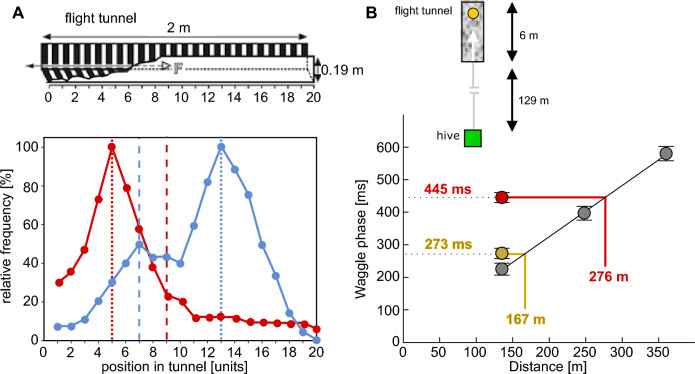
Flight distance learning on outbound and/or inbound flight. **A **Search locations of stingless bees (Melipona) after manipulation of the flight distance between outbound flight to the feeder and the inbound flight back to the tunnel entrance. Red dots: Bees were trained in a textured flight tunnel (above inset) to a food source at position 9 (red dashed vertical line); while taking the reward, the feeder was displaced to position 5, i.e. to a position closer to the tunnel entrance (red dotted vertical line). The search distribution of the bees in the tests at the subsequent foraging trip centred around tunnel position 5, where the feeder was located when the bees started their return flight during the training phase. Blue dots: Bees were trained in a textured flight tunnel to a food source at position 7 (blue dashed vertical line) and displaced during food uptake to position 13, i.e. to a position at a larger distance to the tunnel entrance (blue dotted vertical line). The search distribution of bees during the test phase has two peaks, a prominent peak at position 13, i.e. the feeder position at the start of the inbound flight back to the tunnel entrance, and a smaller peak at position 7, i.e. where the feeder was located at the outbound flight during training (modified from Hrncir et al. 2003). **B **Mean waggle phase durations (± S.E.M.) of dances of honeybees elicited by outdoor feeders at three different distances to the hive (grey circles), with the straight line representing the linear regression on the data. When the bees had to fly the last metres in a textured flight tunnel at a distance of 129 m from the hive, the flight distance indicated by the mean waggle phase durations (± S.E.M.) was largely increased if the bees had to fly through the tunnel at their outbound flight to the food source (red dot and red lines). In contrast, the indicated flight distance was only slightly increased, if the bees had to pass the tunnel on their inbound flight back to the hive (ochre dot and ochre lines) (modified from De Marco and Menzel 2005)

In a detailed study on stingless bees (*Melipona*), the bees experienced during the learning phase either a shorter or longer return flight compared to the preceding outbound flight to a food source during the learning phase. This was accomplished by relocating the feeder, along with the bee, to a different position within the flight tunnel while the bee was still feeding on the rewarding sugar solution. To prevent the bees from perceiving image motion during displacement, they were shielded from visual cues. In the subsequent test, the location of the unavailable food source was assessed. This experimental design allowed for the assessment of the relative contributions of outbound and return flight distances to the bees’ overall distance estimation (Hrncir et al. 2003). When the bees experienced a shorter distance on their return flight during the learning phase, they searched for the feeder around this shorter distance during their subsequent outbound flights, suggesting that they learned the feeder distance on the return flight (Fig. 7A, red dots). Conversely, when the bees encountered a longer distance on the return flight than on the outbound flight during the learning phase, two distinct search peaks were observed in later foraging trips: a larger peak at the location corresponding to the feeder’s position during the return flight and a smaller peak at the feeder’s original location on the outbound flight (Fig. 7A, blue dots). These results suggest that flight distance information is primarily learned during the return flight, though some information is already acquired during the outbound flight to the food source.

These results are only partially consistent with those obtained in similar studies on honeybees (Srinivasan et al. 1997, 1998). Instead of relocating the bees along with the rewarding feeder within the tunnel between the outbound and return flights, the tunnel was extended between these flight segments while the bees were collecting food at the feeder during the learning phase. By manipulating the return and outbound distances during training in this way, the bees searched for the reward during the subsequent test at a distance from the tunnel entrance that matched their experience from the outbound flight in the original (i.e., not elongated) tunnel during learning. This result suggests that bees learn flight distance information during the outbound journey to a food source. However, these results are only partially comparable to those of Hrncir et al. (2003), as Srinivasan et al. (1997, 1998) investigated only the scenario in which the flight tunnel between the outbound and return flights was extended during the training phase. They did not consider the scenario of a shortened flight tunnel, which led Hrncir et al. (2003) to conclude that, in such cases, learning occurs during the return flight.

Further evidence for the learning of flight distance information during the outbound flight to a food source was provided under outdoor conditions (De Marco and Menzel 2005). Honeybees were made to fly either on the outbound flight or, alternatively, on the return flight through a structured tunnel where they encountered a strong optic flow. Unlike the studies discussed above, these experiments analysed the waggle dance as an indicator of learned flight distance, which was first calibrated by correlating the duration of its waggle phase with the distance to various food sources in the environment where the experiments were conducted (see Sect. Bees utilize distance and compass information for navigation). When the flight tunnel was aligned with the direct route between the hive and the food source and when the bees flew through the tunnel on the outbound flight, the waggle phase was much longer than it would have been without the tunnel (Fig. 7B, red dots and lines). However, when the bees passed through the tunnel on the return flight, there was only a minor increase in the signalled flight distance, although the experienced strength of optic flow was similar to the outbound flights (Fig. 7B, ochre dots and lines). These results strongly suggest that the distance information signalled through the waggle dance is primarily determined during the outbound flight to the food source, rather than during the return flight, at least under the conditions of this experiment.

However, displacement experiments in an unfamiliar environment indicate that honeybees may also use the waggle dance to signal the direction of an unknown release site after returning to the hive for the first time. The dances were then consistently aligned with the release site, suggesting that the bees likely acquired directional information during their return flight (Edrich 2015). However, these experiments did not examine the distance information encoded in the waggle phase of the dance. Understanding distance information is particularly important, as bees displaced to an unfamiliar location may require significantly more time to return to the hive than under normal conditions, potentially indicating difficulties in navigating a direct route (Edrich 2015). Nevertheless, because the bees indicated the correct angle to the release site, they seem to have engaged in some level of path integration during the return flight. To support this suggestion, further refinement of these experiments is needed, including systematically tracking bee flight paths and analysing distance information conveyed in the waggle dance.

These studies examining the phase of foraging behaviour during which distance information is learned reveal inconsistencies in the findings (Table 1). These may stem from the different indicators used to assess learned flight distance, i.e. the duration of the waggle phase versus the search behaviour for the location of the food source. Species differences might also contribute, despite the many behavioural similarities between stingless bees and honeybees. Nevertheless, the available evidence supports the conclusion that bees can learn flight distances during both the outbound flight from the hive to a food source and the return flight. The specifics of what is learned during each phase likely depend on the situational context.

## Eye regions and optic flow integration for estimating flight distance

Insects have nearly panoramic visual fields. Since optic flow can vary significantly depending on the spatial layout of the environment viewed by different regions of the eye, an important question emerges: which parts of the visual field are utilized for estimating flight distance based on optic flow? The translational optic flow required for distance estimation can only be reliably analysed in regions of the visual field where environmental structures are sufficiently close to the flying insect (see Sect. Optic flow and spatial information: ambiguities and predictions for flight distance estimation). Moreover, for geometrical reasons, retinal velocities caused even by nearby objects are low in the frontal visual field during forward flight. Therefore, the lateral and ventral visual fields—those that provide views perpendicular to the flight direction on ground structures and the environment below the horizon—are likely to play a more significant role. This aspect, critical from a functional perspective, has been explored in flight tunnels, where the search behaviour for the location of the food source serves as an indicator of flight distance.

**Fig. 8:**
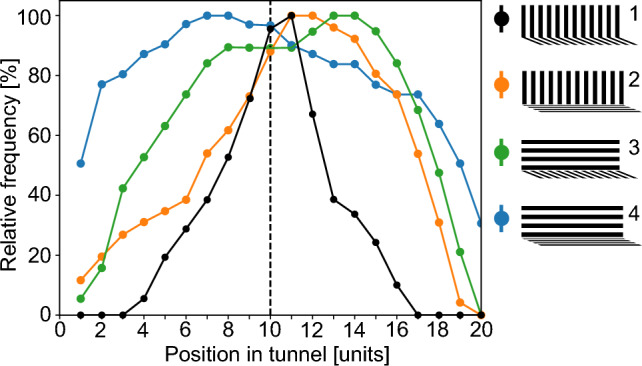
Eye regions used for estimating flight distance. To examine the possibly different role of lateral and ventral optic flow in flight distance estimation, bees were trained to a food source inside differently patterned flight tunnels at position 10 (dashed vertical line). The tunnels differed in the amount of optic flow generated in the lateral and the ventral parts of the visual field. This was achieved by covering the tunnel surfaces with either stripes, aligned along the length of the tunnel, or stripes, aligned perpendicular to the tunnel’s long axis. (1) When bees were trained with strong optic flow induced by both the side walls and floor of the tunnel, in the subsequent tests without food source the search distribution was centred around the position where the food source was previously located (black dots). (2) When bees were trained in a flight tunnel with strong optic flow induced by only the side walls, they searched in approximately the range where the feeder was placed in the training situation (orange dots); however, the search distribution was broader compared to the situation where strong optic flow was induced by walls and floor (green dots). (3) When the bees were trained with strong optic flow induced only by the floor, the search range was considerably larger compared to both previous conditions. (4) When bees were trained to a food source in a tunnel entirely lined with stripes along the length of the tunnel, their search range was much larger than under any other tested condition (blue dots). Obviously, the bees were unable to localize the original position of the food source because the stripes did not provide enough optic flow. The bees need both, lateral and ventral image motion cues to exactly gauge flight distance flown. When lacking the information on either one of these they estimate the distance much less precisely (modified from Hrncir et al. 2003)

To investigate whether lateral and ventral optic flow are used differently by bees to determine their flight path and locate a food source, bees were trained in flight tunnels with side walls and floors that induced either strong or reduced optic flow. This was achieved by using wall textures with stripes oriented either orthogonally or parallel to the main flight direction. The studies, conducted with both stingless bees and honeybees, led to overall consistent results but with quantitative differences in the roles of lateral and ventral visual fields. When optic flow was induced in both the lateral and ventral visual fields during the learning phase, the bees tended to search around the location of the food source during the test phase. The search area was significantly larger when there was reduced optic flow in in part of the visual field. In stingless bees, this effect was observed with reduced flow in both the lateral and, to a lesser extent, the ventral eye regions (Fig. 8) (Hrncir et al. 2003). In contrast, honeybees appeared to rely predominantly on the lateral visual field (Srinivasan et al. 1997).

These two laboratory studies agree that the estimation of distance travelled by bees, when searching for a goal location, is most likely based on both the lateral and ventral visual fields, with the lateral visual field appearing to be more predominant (Table 1). However, the extent to which these findings can be generalized to more natural flight environments and larger spatial scales remains unclear. It is important to note that detectable optic flow with significant retinal velocities occurs in the lateral eye regions primarily when the bee is flying through a cluttered environment, such as among flowers, bushes, or trees that are relatively close. In contrast, in open terrain, sufficient optic flow is available mainly in the ventral visual field, provided the flight altitude is not excessively high. This suggests that the ventral visual field may be important for flight distance estimation based on optic flow, particularly in less cluttered environments. However, to the best of our knowledge, no studies have yet addressed this issue. To address this issue, further studies are needed to systematically manipulate optic flow in different regions of the bee’s visual field. For example, experiments could utilize flight tunnels, similar to those in a previous study on flight speed regulation, with varying widths and heights (Linander et al. 2017). In such setups, bees flying near the centre of a wide tunnel would experience a wide range of optic flow strengths in the lateral visual field, allowing for targeted investigation of how different visual regions contribute to flight behaviour.

## Are distance and direction estimates integrated into a correct goal vector?

Although bees can clearly estimate their flight direction and distance to locate goals (see Sect. Bees utilize distance and compass information for navigation), this ability does not necessarily imply that they are capable of path integration. Path integration requires not only combining estimated path distances with corresponding path directions to form vectors but also integrating these vectors to an overall goal vector along potentially complex and winding routes. Separating these two conceptual steps of the integration process through experimental analysis is not straightforward.

The question of how distance and direction information are integrated has been approached already in the seminal field studies by von Frisch and his colleagues, who used detour experiments to investigate how the waggle dance encodes direction and distance information (von Frisch 1967; Dyer 2002). When foraging bees were led around obstacles, such as a protruding ridge, buildings or forest edges, separating the food source from the hive, their waggle dance indicated the direction of the direct path to the goal, despite the bees had taken a detour. However, the distance communicated by the waggle dance reflected the length of the detour rather than the direct path to the goal (von Frisch 1967). This suggests that while bees may communicate the direction of an untravelled shortcut to the goal, they report the distance based on their actual route. Hence, the corresponding goal vector reflects a fictive performance-dependent location rather than the true location of the goal.

These observations were confirmed in a series of studies conducted both in laboratory settings and in the field (De Marco and Menzel 2005; Evangelista et al. 2014). In these experiments, flight tunnels were used to manipulate the flight conditions in order to mimic a situation corresponding to the so-called triangle completion task, which has been used in numerous experiments with humans and animals to investigate the ability to integrate paths (Zhao and Warren 2015; Jetzschke et al. 2017; Scherer et al. 2024). Subjects are guided along two sides of a triangle forcing them to make a turn and then find their way back to the starting point. The information available for determining the direction and distance of movement varies depending on the specific research question. In the bee experiments based on the triangle completion task, the directional information was provided by the polarisation pattern of the sky, while the distance information was based on the optic flow. The waggle dance was used as an indicator of the goal vector communicated to other bees. These studies, however, did not investigate how the dancing bees themselves used the goal vector in subsequent flights, nor how the recruited bees utilized it.

**Fig. 9:**
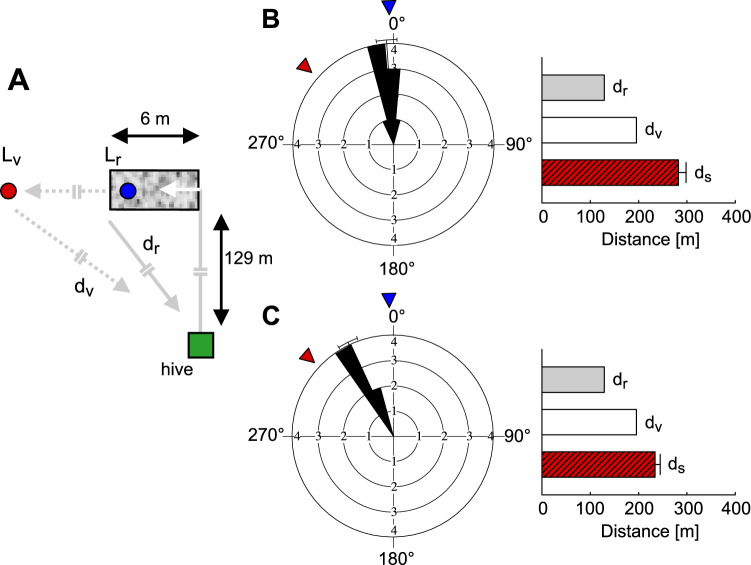
Assessment of how flight distance and direction estimates are integrated. **A **Individually marked bees were trained to forage on a feeder placed at the end of a textured flight tunnel (blue dot) inducing strong optic flow on the eyes, located 129·m away from the hive (green square). The tunnel was oriented 90° to the flight path between its entrance and the hive. The flight distance estimate inside the tunnel as based on optic flow perception was calibrated based on the data shown in Fig. 7B; as a consequence of the strong optic flow induced in the tunnel this virtual distance (d_v_) is much larger than the actual tunnel length of 6 m; the real location of the tunnel exit is denoted by L_r_, the virtual location (red dot) based on the optic flow-based distance estimate by L_v_. The lengths of corresponding goal vectors according to the real location and the virtual location of the tunnel exit are denoted by d_r_ and d_v_, respectively. **B **Estimates of direction and distance based on the waggle dance of bees that had no experience of flying in the tunnel but were familiar with the natural surroundings. Whereas the direction estimate is based on the orientation of the real location of the tunnel exit (blue triangle) in the already known environment (left diagram), the signalled flight distance estimate (d_s_, red bar) reflects the summated optic flow-based distance estimates and, thus, is much larger than both the distance between the hive and the virtual (white bar) and real goal location (grey bar), respectively. **C **Direction and distance estimates based on the waggle dance of bees that had previously foraged inside the flight tunnel while it was aligned with the path between its entrance and the hive (see Fig. 7B). Experienced bees signal a direction  estimate that is significantly shifted towards the virtual target direction (red triangle). The signalled flight distance estimate to the goal (d_s_) is still larger than the correct virtual distance (d_v_) but shorter than the corresponding distance estimate of inexperienced bees. (modified from De Marco and Menzel 2005)

In an outdoor study (De Marco and Menzel 2005), the bees were trained to fly to a food source at the end of a textured flight tunnel. They first had to fly to the entrance of the tunnel, corresponding to one side of the triangle in the triangle completion task. The second segment of the flight, and thus the second side of the triangle, consisted of navigating through the flight tunnel, which was oriented at a 90° angle to the first flight segment (Fig. 9A). Because the tunnel walls were textured, a strong optic flow was induced. Accordingly, the distance signalled by the waggle dance was significantly greater than the real distance (Fig. 9B, right panel). Since the tunnel was open at the top, allowing the bees to use the polarization pattern of the sky to determine their flight direction, the question arises: does the waggle dance indicate the goal direction, corresponding to the length of the two flight segments as estimated from the optic flow measurements? For naïve bees encountering the tunnel situation for the first time, this was not the case. They signalled the length of the flight path indicated by the optic flow via their waggle dance. However, they did not seem to account for the longer flight distance indicated by the optic flow when estimating and signalling the goal direction (Fig. 9B, left panel). However, as the bees became more familiar with the tunnel situation, they began to signal a direction by their waggle dance that more closely matched the goal direction as provided by the optic flow-based distance information, although it still did not fully align with the signalled distances (Fig. 9C, left panel). The signalled flight distance was still longer than the actual shortcut vector between the virtual position of the food source and the nest, but shorter than the corresponding distance signalled by inexperienced bees (Fig. 9C, right panel). This suggests that, under these conditions, at least inexperienced bees are unable to combine directional information with the optic flow-based distance information obtained along the individual path segements to form the correct goal vector. However, it seems that with increasing experience of the specific environment, at least the direction of the goal vector, signalled by the waggle dance, is improved (De Marco and Menzel 2005).

**Fig. 10:**
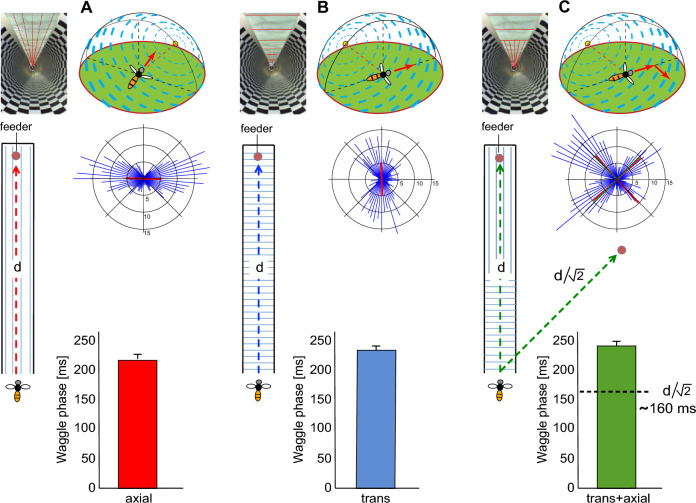
Assessment of how flight distance and direction estimates are integrated. Direction estimates signalled by the waggle dance based on orientation of polarization pattern above a flight tunnel. Bees were trained to a food source at the end of a textured flight tunnel with either axially oriented polarized illumination (**A**), with transversely polarized illumination (**B**), or with transversely polarized illumination in the first half and axially polarized illumination in the second half of the tunnel simulating a path along the equal sides of a right-angled triangle (**C**). The orientation of the polarisation pattern is indicated by the lines in the photographic view into the flight tunnel (top left in **A**–**C**) and in the schematic view of the tunnel (middle left in **A–C**). The orientation of the bee relative to the sun indicated by the polarisation is shown at the top right for each polarisation pattern. The directions signalled by the waggle dance scatter around the corresponding directions simulated by the polarization pattern (circular plots in ** A**–**C**). Note that for the simulated triangle, the direction appears to reflect the vector sum of the vectors corresponding to the two sides of the triangle. Since the polarisation patterns are the same for directions of movement that differ by 180°, the directions signalled by the waggle dance are also ambiguous. **A–C** bottom diagrams: Mean waggle phase durations of the dances corresponding to the different polarisation conditions. The waggle phase durations and, thus, the signalled flight distances do not differ significantly for any of the polarisation conditions. This finding implies that also under the condition where two sides of a right-angled triangle are mimicked, the absolute flight distance and not the short-cut distance to the hive, i.e. the length of the goal vector (horizontal dashed line) is signalled by the waggle dance (modified from Evangelista et al. 2014)

The conclusion that the estimated flight distance, at least in an unfamiliar situation for the bee, is not geometrically correctly linked to the corresponding flight direction was further supported by a laboratory study with precisely controlled environmental conditions (Evangelista et al. 2014). Bees were trained to fly to a food source located at the end of a textured tunnel illuminated from above with light polarized either parallel or perpendicular to the axis of the tunnel (Fig. 10A, B). The bees used their waggle dance to signal directions corresponding to the polarized light patterns, which were orthogonal to each other. This indicated that the bees utilized the polarized light information to signal directions as they might in a natural flight situation. When the illumination in the first half of the tunnel was polarized axially and in the second half transversely, simulating an L-shaped tunnel and, thus two sides of a right-angled triangle, the bees danced along the main diagonal directions (Fig. 10C). The bees thus seem to perceive the two segments as orthogonal to each other although they did not have to fly a turn, and proprioceptive information would have signaled a straight flight. This finding suggested that the bees according to the polarization information were signalling the correct direction of the goal vector, as both sides of the simulated triangle were of equal length, covered with the same texture, and thus induced approximately the same amount of optic flow. Nevertheless, the bees communicated the total distance flown with the duration of their waggle phase, rather than the length of the goal vector corresponding to the imaginary shortcut in the signalled direction (Fig. 10C) (Evangelista et al. 2014). This again showed that the information conveyed by the waggle dance did not align with the geometric predictions of path integration. However, unlike the combined field/tunnel experiments described above (Fig. 9), the bees had no opportunity to test and refine the direction and distance information acquired during their outward flight when returning to the hive because they were required to return through the same flight tunnel.

**Fig. 11:**
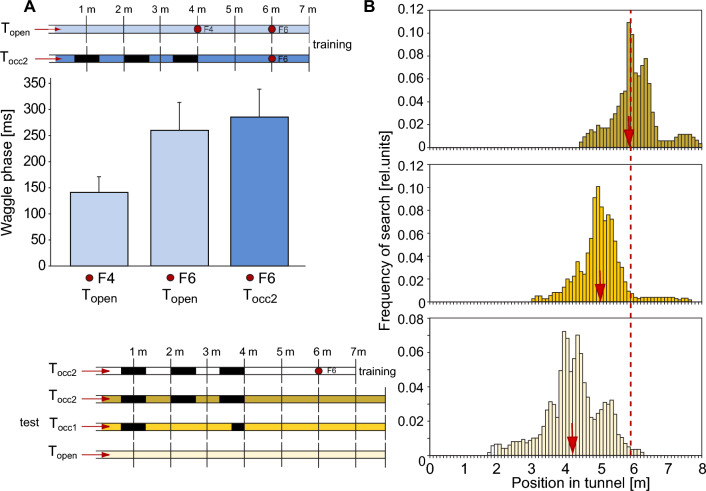
Influence of celestial polarisation cues on the flight distance indicated by both the waggle dance and search location. **A **Waggle dance as an indicator of estimated flight distance: Bees were trained to forage either in an open flight tunnel with a view of the sky polarisation pattern, to food sources placed at 4 m (F4) or 6 m (F6) from the tunnel entrance (T_open_) or they were trained to a food source 6 m from the tunnel entrance (F6), with 2 m of the tunnel ceiling occluded, so that the bees could see the polarisation pattern of the sky only for a total of 4 m of the tunnel (T_occ2_) (see inset above; the colours in the inset correspond to the tunnels that were used to analyse the corresponding waggle dances). When trained in T_occ2_, the duration of the waggle phase (right column) was significantly longer than for a food source at 4 m, signalling a distance to the food source like at F6 in T_open_. The flight distance that bees signal to other bees through their waggle dance is therefore only dependent on the distance flown, regardless of whether the bee could see the polarisation pattern of the sky. **B **Search location as an indicator of estimated flight distance: Bees were trained to fly to a food source 6 m from the entrance (F6) of a 7 m long tunnel. The polarisation pattern of the sky was occluded by three panels for a total of 2 m (T_occ2_). The effect of celestial polarisation input on the location at which bees search for a previously learned feeder was tested in three 8 m flight tunnels without a food source but with varying amounts of open sky, i.e. with a total of 2 m (T_occ2_) or 1 m (T_occ1_) occluded or no occlusion at all (T_open_) (see inset at bottom left; the colours of the schematic flight tunnels match those of the corresponding search histograms). When tested in a tunnel with a covering configuration identical as used during training (T_occ2_), the mean search location was not significantly different from the feeder location during training. When tested in a tunnel in which the occlusion of celestial polarisation was reduced by 1 m (T_occ1_) compared to the training situation (T_occ2_), the mean search location was shifted to the tunnel entrance by approx. 1 m. When tested in a fully open tunnel (T_open_), the mean search location was approximately 2 m closer to the entrance than in the training situation. The distance at which bees search for a previously learned food source depends on whether they have polarisation information or not. The bees only used flight distances for which information about the celestial compass was available (modified from Dacke and Stinivasan 2008)

These studies have utilised the waggle dance to indicate the learned flight direction and distance, thereby conveying foraging information to other bees. Complementing this, it is equally important to examine where the dancing bees themselves search for the food source on their next foraging trips. One study specifically investigated the influence of celestial compass information on both the flight distance signalled by the waggle dance and the search location, but only along a straight path without mimicking a triangle completion task (Dacke and Srinivasan 2008). In this study, bees were trained in a flight tunnel in which the polarization pattern of the sky was either present or partially occluded; the proportion of the tunnel in which the polarization pattern was occluded or could be seen was systematically varied between the learning and test phases (Fig. 11A). The results showed that the waggle dance encodes information about the total distance flown to the food source, even if celestial compass information was only available for part of the journey. This suggests that, in this experimental setting, there is no direct link between the measured flight distance and the celestial compass information. However, when returning to a learned food source location, bees used only the estimated flight distance corresponding to the flight segments for which celestial compass information was available during training (Fig. 11B) (Dacke and Srinivasan 2008). The flight distance that bees signal to other bees through the waggle dance thus differs from the distance they use when searching themselves for a food source, depending on the availability of celestial compass information. Based on these experiments, Dacke and Srinivasan (2008) propose that honeybees use two types of odometric memories depending on the context. For reporting the distance flown to nestmates through the waggle dance, a ‘community’ odometer is assumed to integrate optic flow information along the entire route, regardless of sky visibility. For navigating back to a previously visited food source, the ‘personal’ odometer incorporates celestial compass information. At present, the potential functional significance of having two different odometric memories remains speculative.

It should be noted that the experimental approach of Dacke and Srinivasan (2008) focused exclusively on the estimation of flight distances and the relevance of celestial information, without investigating whether and how flight distance is combined with directional information to determine a goal vector. Although the waggle dance data suggest that optic flow-driven flight distance estimation is decoupled from directional information processing at least when flying in unfamiliar terrain, the question of how bees use these variables themselves to find previously learned goals remains unresolved (Table 1). To our knowledge, no studies have investigated this integration for Hymenoptera in flight, in contrast to research on walking insects (see Sect. Introduction).

## Conclusions: path integration-is it a ubiquitous ability in Hymenoptera?

Path integration is widely regarded as essential for navigation in unfamiliar environments, in particular, for locating previously learned goals. Evidence from studies on walking insects—primarily ants, but also walking bumblebees and *Drosophila*, for which path integration has been convincingly demonstrated—is often generalized to flying insects. Whether this generalisation is based on sufficiently solid ground has been critically reviewed in this paper based on the relevant literature (Table 1). First, it was necessary to investigate how accurately the distance travelled during flights can be estimated using optic flow, considering the inherent geometrical ambiguities it contains. Second, it was crucial to evaluate whether insects can integrate different segments of their flight into a cohesive goal vector, thereby demonstrating the ability to perform path integration during flight.

In the context of path integration, it is assumed that in insects the optic flow is used as a proxy for the flight speed over ground, which, when integrated over time, provides information about the distance travelled. However, because optic flow depends on the animal’s nearness to objects in the environment—or, in the case of the ventral visual field, on the height above the ground—it only accurately reflects flight speed if the animal can determine these distances through additional means, such as by independently measuring flight altitude (Egelhaaf 2023). Based on current evidence, it can be concluded that bees do not effectively resolve these ambiguities, at least within the context of the behavioural paradigms used for analysis. They appear to lack mechanisms for using additional information, such as flight altitude measurements, to accurately determine flight distance (Bergantin et al. 2021). Consequently, relying solely on optic flow for path integration leads to inaccurate flight distance estimates, as demonstrated in numerous experiments (Sects. Outbound vs. inbound flight: when is distance travelled estimated? and Are distance and direction estimates integrated into a correct goal vector?). Although differences in spatial structure might be averaged out over longer distances, this averaging is less effective over short flight segments, especially in cluttered environments. In such scenarios, proximity to individual objects causes substantial fluctuations in the time-dependent optic flow measurements and thus lead to highly unreliable flight distance estimates (Fig. 5) (Egelhaaf et al. 2014; Schwegmann et al. 2014a, b; Li et al. 2017). These complexities are often not considered in functional models of sensory and neural processing in the central brain underlying path integration.

The consequences of these ambiguities in optic flow for flight distance estimation are not only theoretically predicted but also evident in bees’ waggle dance and their search for previously learned goal locations. Manipulating optic flow by having bees fly through differently textured flight tunnels showed that they significantly misestimate flight distances depending on the strength of the optic flow. These environment-dependent ambiguities in flight distance estimation are not problematic as long as the bee follows a similar route to the goal as she did when learning its location, or if a bee recruited by a waggle dance takes a route that closely matches the one used to derive the distance communicated by the dancing bee. However, if these conditions are not met, such ambiguities in optic flow and the resulting misjudgement of flight distance pose a significant challenge to path integration, given that optic flow is detectable in both the lateral and ventral visual fields for flight distance estimation. (Fig. 4; Sects. Estimating travel distance using optic flow information and Eye regions and optic flow integration for estimating flight distance).

Interestingly, the flight distance information communicated by the waggle dance is continuously updated and consolidated based on experience. This has been demonstrated in experiments that systematically varied the distance to a food source and tracked changes in the waggle phase duration, showing that the information conveyed to other foragers by the waggle dance is gradually refined with repeated flights (Chatterjee et al. 2019). This conclusion is consistent with the results summarised in Sect. Are distance and direction estimates integrated into a correct goal vector? (Fig. 9), which demonstrate that bees familiar with a textured flight tunnel placed in a natural environment used to manipulate optic flow-based flight distance estimates signalled a direction in their waggle dance that more closely matched the actual goal direction compared to inexperienced bees (De Marco and Menzel 2005). It is also consistent with recent findings indicating that bees flying toward a goal through an unfamiliar, structured tunnel generating strong optic flow, while simultaneously observing parts of a familiar natural environment, conveyed a flight distance in their waggle dance corresponding to the familiar environment rather than the current optic flow (Menzel and Galizia 2024). It would be interesting to determine whether, as in the study by Chatterjee et al. (2019), the bees would gradually adapt their waggle dance to reflect the optic flow induced by the tunnel and thereby adjust to the corresponding flight distance as their experience with the tunnel increases.

Path integration involves combining direction and distance information to form a goal vector. As outlined in Sect. Are distance and direction estimates integrated into a correct goal vector?, there is currently no definitive evidence that bees combine the directional information derived from the celestial compass with the flight distance determined from optic flow measurements to create a vector representation of their goal in unfamiliar environments (Table 1). Therefore, it remains unclear whether they can perform path integration during flight to navigate back to their starting point in an unfamiliar environment after a convoluted outbound journey. Although there is convincing evidence that bees can take shortcuts between two familiar locations if they have had the opportunity to get familiar with the environment beforehand (Menzel and Greggers 2015; Menzel 2023), it remains an open question, whether they are able to take such shortcuts in flight based solely on path integration, without relying on previously learned environmental information such as landmarks, the overall visual landscape, or other cues.

In summary, there is currently no conclusive evidence that insects use path integration during flight (Table 1). As discussed, many questions remain unresolved, and more detailed experiments are necessary. Notably, it should be considered that the information communicated to other foragers through the waggle dance may differ from the information the bee uses during her own search for a food source (Dacke and Srinivasan 2008; Chatterjee et al. 2019). Such a difference was hypothesised as a potential factor in updating the learned flight distance information. Whereas the updating of stored flight distance information conveyed by the waggle dance was concluded to rely on the gradual incorporation of previously learned spatial information, the bee’s own foraging was hypothesized to be more influenced by the current distance information (Chatterjee et al. 2019). The extent to which these conclusions and the various analyses reviewed here align to form a coherent understanding requires further investigation. Conducting such experiments is considerably more challenging with flying insects than with walking ones, which likely explains the lack of existing studies. Therefore, we should be cautious in extrapolating the results of path integration observed in walking insects to those that fly.

Given the lack of clear evidence for genuine path integration in flying insects, we will end by looking at path integration in flying insects from a complementary perspective. Why would path integration be of crucial importance for flying insects at all, especially for central place foragers like honeybees, stingless bees, and bumblebees, which usually navigate in environments rich in spatial structures like flowers, undergrowth, bushes, and trees? Path integration was initially studied systematically in desert ants, which must navigate barren landscapes devoid of visual landmarks. In such environments, ants rely heavily on path integration to efficiently find their nest after foraging in harsh conditions. The selective pressure for reliable path integration in these relatively featureless habitats is strong, and evidence suggests that it functions with remarkable accuracy (Wehner 2020). It is interesting to note that ant species living in spatially more structured environments rely less on path integration and instead make greater use of visual environmental features, such as the surrounding panorama and prominent landmarks near the nest, to navigate back to the nest after a foraging trip (Cheng et al. 2012; Narendra et al. 2013; Buehlmann et al. 2016). Hence, a key feature of the hymenopteran navigation system is its redundancy, enabling these insects to select from a toolbox of navigation mechanisms the ones most appropriate for the specific context.

Accordingly, as the environments of flying Hymenoptera are typically well-structured with ample visual landmarks, foragers engage in characteristic orientation behaviour during their initial flights from the nest, starting with learning/exploration flights within a limited spatial range that gradually expands. Although the details of what is learned during these flights remain unclear, it is evident that they are critical for later foraging efficiency (Lehrer 1993; Lihoreau et al. 2012; Zeil 2012; Degen et al. 2015, 2016; Stürzl et al. 2016; Woodgate et al. 2016; Collett and Zeil 2018; Lobecke et al. 2018; Bertrand and Sonntag 2023; Collett and Hempel de Ibarra 2023). Displacement experiments suggest that even a single exploration flight may suffice to enable bees to undertake a relatively direct return flight during subsequent foraging trips (Degen et al. 2016). During these exploration flights, visual environmental features might be linked with distance and directional information through output signals of central complex circuits. Such mechanisms have been demonstrated in ants, whose homing behaviour and route learning have been modelled extensively. While these models differ in detail, they are rooted in experimental evidence about the roles of the central complex in path integration and the mushroom bodies in visual learning (Ardin et al. 2016; Hoinville and Wehner 2018; Webb 2019; Le Möel and Wystrach 2020; Bennett et al. 2021; Wystrach 2023; Clement et al. 2024; Jesusanmi et al. 2024; Steinbeck et al. 2024). Familiarity with the visual environment is particularly critical for guiding navigation back to a home location. One specific model posits that parallel, lateralized visual memories are acquired continuously as the animal explores a novel environment. During the learning phase, the mushroom bodies are assumed to encode ‘left’ and ‘right’ visual memories, reinforced by signals from the central complex that indicate deviations from the goal direction (left or right). When navigating back, these memories generate control signals based on the familiarity of the visual input, guiding the animal toward its goal (Wystrach 2023). This approach emphasizes that circuits involved in path integration also facilitate visual learning ‘internally’ (Wystrach 2023).

However, all these modelling studies assume that navigation occurs on the ground and, critically, that the distance travelled is precisely determined. As highlighted in this review, such assumptions cannot be generalized to flight. Therefore, it is essential to systematically investigate the extent to which these models can also explain navigation in three-dimensional (3D) space. Particular attention should be given to their robustness in addressing ambiguities arising from optic flow-based flight distance estimates, which can vary depending on the spatial layout of the environment and the flight altitude. Because honeybees perform exploratory flights to familiarise themselves with their surroundings before foraging, and because they initially fly relatively close to the nest and in specific sectors around it (Degen et al. 2015, 2016), it may be assumed that the ambiguities of the optic flow-based distance information will not have too strong an effect, especially as the animals then return to the nest in approximately the same environmental sector. If the direction and distance information obtained in this way is linked to visual memories of environmental structures in the ventral and, depending on the spatial structure of the environment, lateral visual field, relatively direct return flights to the nest are conceivable on this basis, with distance estimates improving iteratively through repeated trips. Interestingly, exploratory flight orientation seems to be guided, in part, by prominent environmental features, further simplifying finding home (Degen et al. 2015). As the spatial range of these flights expands progressively before the foraging phase, it is plausible that direction and distance information become iteratively linked to visual environmental cues for increasingly larger distances. This might enable bees to find their way back to the hive over longer distances, despite the inherent ambiguities of optical flow-based distance estimation.

We conclude that by iteratively integrating multiple optic flow-based distance estimates, the accuracy of the estimated distance to a goal location can be progressively improved. Honeybees can then use this refined information to communicate relatively precise location details to other foragers through the waggle dance. However, as discussed in this review, evidence suggests that flight vectors derived from celestial direction cues and optic flow-based distance measurements are only reliably effective for navigation within sufficiently similar spatial environments, due to the inherent ambiguities of optic flow. Nonetheless, these flight vectors remain integral to localization and navigation, even in the absence of true path integration. When associated with environmental views, they may contribute to robust and reliable navigation strategies in visually rich environment (Zeil 2022).

## Data Availability

No datasets were generated or analysed during the current study.
